# Establishment of hairy root cultures of *Lactuca serriola* and enhancement of phenolic metabolites through methyl jasmonate elicitation

**DOI:** 10.1371/journal.pone.0360818

**Published:** 2026-11-04

**Authors:** Maisa Asheri, Alireza Farokhzad, Mohammadreza Naghavi, Raheleh Ghasemzadeh, Pejman Azadi, Meisam Zargar

**Affiliations:** 1 Department of Horticultural Science, Faculty of Agriculture, Urmia University, Urmia, Iran; 2 Division of Biotechnology, Department of Agronomy and Plant Breeding, College of Agricultural and Natural Resources, University of Tehran, Karaj, Iran; 3 Department of Agrobiotechnology, Institute of Agriculture, RUDN University, Moscow, Russia; 4 Department of Plant Production and Genetics, Faculty of Agriculture, Urmia University, Urmia, Iran; 5 Department of Genetic Engineering, Agricultural Research, Education and Extension Organization (AREEO), Agricultural Biotechnology Research Institute of Iran (ABRII), Karaj, Iran; University of Kotli, PAKISTAN

## Abstract

*Lactuca serriola* is a wild lettuce species that synthesizes diverse phenolic compounds involved in plant defense and adaptation to environmental stress. Methyl jasmonate (MeJA), a key signaling molecule in plant defense, is known to regulate secondary metabolism by activating the phenylpropanoid pathway. In the present study, genetically stable hairy root cultures of *L. serriola* were established to investigate MeJA-induced metabolic reprogramming and its effects on phenolic accumulation and antioxidant defense responses. Hairy roots were induced using *Agrobacterium rhizogenes* strain ATCC 15834 and molecularly confirmed by PCR amplification of the *rolC* gene. The selected fast-growing hairy root line was treated with 0, 100, and 200 μM MeJA. MeJA significantly affected both growth and secondary metabolism. Although increasing MeJA concentrations reduced fresh and dry biomass, they promoted the accumulation of total phenolics and flavonoids. HPLC-DAD analysis further demonstrated significant increases in several phenolic metabolites, including caffeic acid, chlorogenic acid, *p*-coumaric acid, rosmarinic acid, and cinnamic acid. MeJA treatment also enhanced the activities of superoxide dismutase, catalase, ascorbate peroxidase, and guaiacol peroxidase, indicating activation of antioxidant defense systems associated with jasmonate-mediated stress responses and phenylpropanoid metabolism. This study provides the first comprehensive characterization of MeJA-induced phenolic metabolism in *L. serriola* hairy roots and demonstrates that MeJA effectively reprograms secondary metabolism toward enhanced phenolic biosynthesis. These findings advance our understanding of jasmonate-regulated defense metabolism in hairy roots while supporting the use of this *in vitro* system as a controlled platform for future studies and potential biotechnological production of specialized metabolites.

## 1. Introduction

*Lactuca serriola* L. (prickly or wild lettuce), a member of the Asteraceae family, is the wild progenitor of cultivated lettuce (*Lactuca sativa* L.) [1]. Closely related species such as *L. sibirica* and *L. indica* share overlapping morphological and phytochemical characteristics [2,3]. *L. serriola* synthesizes a wide range of secondary metabolites, including phenolic acids (e.g., trans-*p*-coumaric acid, protocatechuic acid, and caffeic acid), flavonoids (e.g., isoquercitrin and luteolin), triterpenoids, and steroids [4,5]. These metabolites contribute to the plant's antioxidant capacity and play important roles in defense against environmental stresses, pathogens, and herbivores [6–9]. The characteristic milky latex (lactucarium) further represents an important defensive feature of the genus.

Secondary metabolites are integral components of plant adaptive responses to environmental stimuli. Their biosynthesis is regulated by developmental cues as well as biotic and abiotic stresses, enabling plants to maintain cellular homeostasis and survive unfavorable conditions [10]. Among these metabolites, phenolic compounds constitute one of the largest and most diverse groups [11,12]. Phenolic acids participate in reactive oxygen species (ROS) scavenging, reinforcement of cell walls, ultraviolet protection, and defense signaling, thereby contributing to plant growth, development, and stress tolerance [13,14]. Understanding the regulation of phenolic metabolism is therefore important for elucidating plant adaptive mechanisms under stress conditions.

Because phenolic biosynthesis is strongly influenced by environmental factors, controlled *in vitro* culture systems provide valuable experimental platforms for investigating the regulation of secondary metabolism [15]. Hairy root cultures induced by *Agrobacterium rhizogenes* exhibit rapid growth, extensive lateral branching, and remarkable genetic and biochemical stability while maintaining the biosynthetic capacity of differentiated roots. These characteristics make hairy roots particularly suitable for studying metabolic regulation and plant responses to elicitor treatments under highly controlled conditions [16].

Among the elicitors commonly employed in plant biotechnology, methyl jasmonate (MeJA) is one of the most effective for activating plant defense responses. As an important signaling molecule in jasmonate-mediated pathways, exogenous MeJA regulates numerous physiological and molecular processes associated with stress adaptation [17,18]. One of its major effects is activation of the phenolic biosynthesis through the induction of key biosynthetic genes, including phenylalanine ammonia-lyase (PAL) and chalcone synthase (CHS), leading to enhanced accumulation of phenolic acids and flavonoids [19]. Consequently, MeJA has become a widely used experimental tool for investigating the regulation of secondary metabolism in plant tissue cultures.

The phenolic acids investigated in the present study, including caffeic acid, chlorogenic acid, *p*-coumaric acid, rosmarinic acid, and cinnamic acid, are synthesized through the phenylpropanoid pathway via sequential hydroxylation, methylation, and conjugation reactions. For example, rosmarinic acid is formed through the condensation of 4-coumaroyl-CoA and 4-hydroxyphenyllactic acid, catalyzed by rosmarinic acid synthase (RAS), followed by subsequent hydroxylation reactions [20,21]. These compounds contribute substantially to the plant antioxidant defense system and enhance the ability of plants to cope with oxidative stress.

Previous studies have demonstrated that MeJA effectively stimulates phenylpropanoid metabolism in hairy root cultures of diverse plant species. In *Salvia miltiorrhiza*, treatment with 100 µM MeJA significantly increased the accumulation of rosmarinic acid and lithospermic acid B [22]. Similarly, MeJA enhanced the production of rosmarinic acid, chlorogenic acid, caffeic acid, and cinnamic acid in *Mentha spicata* hairy roots [23]. Although these findings demonstrate the conserved role of jasmonate signaling in regulating secondary metabolism, comparable studies have not yet been conducted in *L. serriola* hairy root cultures.

To date, no comprehensive study has examined the coordinated effects of MeJA on phenolic metabolism and antioxidant enzyme responses in *L. serriola* hairy root cultures. The present study addresses this gap by evaluating MeJA-induced changes in phenolic acid and flavonoid accumulation together with the activities of key antioxidant enzymes (SOD, CAT, GPX, and APX). By integrating metabolite profiling with biochemical analyses, this work provides new insight into jasmonate-mediated regulation of phenylpropanoid metabolism and antioxidant defense in transformed roots, thereby improving our understanding of stress-responsive specialized metabolism in *L. serriola*.

## 2. Materials and methods

### 2.1. Chemicals and reagents

Methyl jasmonate (95%), Folin–Ciocalteu reagent, aluminium chloride, gallic acid, quercetin, and phenolic standards (cinnamic acid, p-coumaric acid, caffeic acid, chlorogenic acid, rosmarinic acid, rutin, apigenin, and quercetin) were purchased from Sigma-Aldrich (St. Louis, MO, USA). Acetonitrile and acetic acid (HPLC grade) were obtained from Merck (Darmstadt, Germany). All other chemicals and solvents were of analytical grade.

### 2.2. Establishment of *Lactuca serriola* hairy root cultures

Seeds of *Lactuca serriola* were gathered from Karaj, Iran (35°46′ N, 51°00′ E). They were surface-sterilized with 70% (v/v) ethanol for 1 min, followed by 5% (v/v) sodium hypochlorite for 10 min, and then rinsed five times with sterile distilled water. Sterilized seeds were germinated on solid MS medium [24] containing MS salts and vitamins, 30 g/L sucrose, and 7 g/L agar (pH 5.7) at 25 °C under a 16/8 h (light/dark) photoperiod.

*Agrobacterium rhizogenes* strain ATCC 15834 was obtained from the National Institute of Genetic Engineering and Biotechnology (NIGEB), Tehran, Iran. The bacterium was cultured overnight in liquid Luria–Bertani (LB) medium (pH 7.0) at 28 °C on a rotary shaker (180 rpm). Bacterial cells were pelleted by centrifugation at 3,000 × g for 10 min and resuspended in liquid MS medium containing 50 g/L sucrose to an OD_600_ of 1.0 for inoculation.

Cotyledonary leaves from 2-week-old aseptic seedlings were used as explants. The explants were wounded and pre-cultured for 2 days in the dark on solid MS medium. Pre-cultured explants were immersed in the bacterial suspension for 5 min, blotted dry, and co-cultivated on hormone-free solid MS medium (7 g/L agar) at 25 °C in the dark. After 2 days of co-cultivation, explants were transferred to half-strength MS (½ MS) medium containing 400 mg/L cefotaxime to eliminate excess bacteria [25].

Hairy roots appeared at the infection sites within 2–3 weeks. Putative transformed roots were excised and subcultured every 2 weeks on hormone-free ½ MS solid medium containing gradually decreasing concentrations of cefotaxime (400 → 300 → 200 → 100 → 50 → 0 mg/L). After complete elimination of bacteria (approximately 2 months), axenic hairy root lines were transferred to 250 mL Erlenmeyer flasks containing 30 mL of hormone-free ½ MS liquid medium and maintained on a rotary shaker at 120 rpm, 25 °C in the dark, with subculturing every 14 days [26].

For selection of the fastest-growing line, 500 mg fresh weight of each stabilised line (after four subcultures) was inoculated onto solid ½ MS medium containing 50 mg/L cefotaxime. After 19 days of culture at 25 °C in darkness, growth index (GI) was calculated according to using the formula [27]:

GI=(final fresh weight−initial fresh weight)/initial fresh weight

The line exhibiting the highest growth index and vigorous lateral branching was selected for all subsequent elicitation experiments.

### 2.3. PCR confirmation of genetic transformation

Genomic DNA was isolated from non-transformed roots, putative hairy root lines, and *Agrobacterium rhizogenes* ATCC 15834 using the CTAB method [28]. The presence of the *rolC* gene (626 bp) was confirmed by PCR to verify stable T-DNA integration.

The primer pairs used were (Table 1):

**Table 1:** Primer pair sequences for *rol C.*

Gene	Primer sequence (5′ → 3′)	Product size
rolC	F: CTCCTGACATCAAACTCGTC	626 bp
	R: TGCTTCGAGTTATGGGTACA	

PCR reactions (25 µL) contained 50–100 ng template DNA, 1 × PCR buffer, 1.5 mM MgCl_2_, 0.2 mM dNTPs, 0.4 µM each primer, and 1 U Taq DNA polymerase. Amplification conditions were: initial denaturation at 94 °C for 5 min; 35 cycles of 94 °C for 45 s, 58 °C for 45 s, and 72 °C for 1 min; and a final extension at 72 °C for 7 min. PCR products were separated on 1.2% agarose gels stained with ethidium bromide and visualized under UV light. A 1 kb DNA ladder was used as molecular size marker. Positive control (*A. rhizogenes* genomic DNA), negative control (non-transformed root DNA), and no-template control were included in each run.

### 2.4. Methyl jasmonate (MeJA) elicitation experiments

Methyl jasmonate (Sigma-Aldrich) was first dissolved in 100% ethanol to prepare a 10 mM stock solution and then sterilized using 0.2 µm syringe filters. Working concentrations of 100 and 200 µM were obtained by adding appropriate volumes of the sterile stock solution (50 µL and 100 µL, respectively) to 50 mL of autoclaved half-strength MS liquid medium (pH 5.7). Actively growing 19-day-old hairy root cultures (log phase) were treated with MeJA with 0 (control), 100, and 200 µM. Hairy root samples were collected 24 and 48 h after elicitation in three biological replicates. The samples were briefly blotted on sterile filter paper to remove surface moisture, weighed, rapidly frozen in liquid nitrogen, and stored at –80 °C until further analysis [29].

### 2.5. Determination of hairy roots biomass

At each harvest time point, hairy roots were separated from the culture medium, gently blotted on sterile filter paper to remove adherent liquid, and fresh weight (FW) was immediately recorded. Dry weight (DW) was determined after the samples were freeze-dried for 48 h.

### 2.6. Extraction and determination of total phenolic and total flavonoid content

Fresh hairy roots (500 mg) were homogenised in liquid nitrogen and extracted with 20 mL of 80% (v/v) aqueous methanol by ultrasonication for 30 min at 30 °C. The extract was centrifuged at 10,000 × g for 15 min at 4 °C, and the supernatant was filtered through Whatman No. 41 filter paper. The resulting filtrate was used for the spectrophotometric assays described below [30].

#### 2.6.1. Total phenolic content (TPC).

Extract (40 µL) was mixed with 120 µL of Folin–Ciocalteu reagent and 40 µL of distilled water. After 5 min, 100 µL of 20% (w/v) sodium carbonate was added, and the mixture was incubated in the dark at 25 °C for 1 h. Absorbance was measured at 750 nm using a microplate reader. TPC was expressed as milligrams of gallic acid equivalents (GAE) per gram of fresh weight (FW) using a gallic acid calibration curve (R^2^ ≥ 0.999) [31].

#### 2.6.2. Total flavonoid content (TFC).

Extract (30 µL) was mixed with 30 µL of 5% sodium nitrite. After 5 min, 60 µL of 10% aluminium chloride was added, followed 1 min later by 180 µL of 1 M potassium acetate. Absorbance was measured at 380 nm after 15 min. TFC was expressed as milligrams of quercetin equivalents (QE) per gram of fresh weight (FW) using a quercetin calibration curve (R^2^ ≥ 0.999) [32].

### 2.7. Extraction and HPLC-DAD analysis of individual phenolic compounds

Fresh hairy root samples (500 mg) were rapidly frozen in liquid nitrogen and homogenized in 5 mL of 80% methanol, which was added immediately to the frozen tissue to minimize oxidative degradation. The homogenates were subjected to ultrasonic extraction (Eurosonic 4D, Euronda, Italy) for three cycles of 20 min at 25 °C. Following centrifugation at 2000 rpm for 10 min (MIKRO 22R, Hettich, Germany), the supernatants were filtered through a 0.22 µm PTFE syringe filter [26].

High-performance liquid chromatography (HPLC) analysis for Separation, identification, and quantitative determination of phenolic acids was carried out using an Agilent 1100 series system equipped with a quaternary gradient pump, online degasser, column oven maintained at 25 °C, a 20 µL injection loop, and a diode-array detector (DAD) set at 250, 272, and 310 nm. Chromatographic separation was achieved on a reversed-phase C18 column (ZORBAX Eclipse XDB-C18; 250 mm × 4.6 mm i.d., 5 µm particle size; Dr. Maisch, Germany). Data acquisition and processing were performed using ChemStation software.

The mobile phase consisted of acetonitrile (solvent A) and aqueous acetic acid (1%, v/v) (solvent B), delivered at a flow rate of 1.0 mL min^-1^. A gradient elution program was applied, starting with 10% A and increasing to 25% A over 5 min, followed by an increase to 65% A within the next 10 min. The total chromatographic run time was 15 min.

Individual phenolic compounds were identified by comparing their retention times and UV spectra with those of authentic standards, and quantification was performed using external calibration curves based on integrated peak areas.

Quantification was based on seven-point external calibration curves (R² ≥ 0.9992). Results were expressed as micrograms per gram of fresh weight (µg/g FW). All samples were analysed in triplicate.

### 2.8. Antioxidant enzyme activity

#### 2.8.1. Preparation of crude enzyme extract.

To prepare samples for antioxidant enzyme activity measurements, the fresh samples were subjected to liquid nitrogen grinding at a dosage of 500 mg each. The powdered samples were combined with a 2 mL of extraction solution (0.5% Tris-HCL, pH 8.0 and 0.05% polyvinyl pyrrolidone). The extract was centrifuged at 13,000 rpm for 20 min at 4°C. The obtained supernatant was utilized to assess the activity of antioxidant enzymes such as catalase (CAT), guaiacol peroxidase (GPX), ascorbate peroxidase (APX), and superoxide dismutase (SOD) activities [33].

#### 2.8.2. Catalase activity (CAT).

Quantitative measurement of catalase activity was performed using the spectrophotometric approach at 240 nm introduced by Aebi (1984) [34]. The reaction mixture contained 3 µL of the extract followed by the addition of 285 µL of phosphate buffer (pH 7.0) and 6 µL of 3% hydrogen peroxide (H2O2). The degree of H2O2 decomposition within 1 min served as the basis for determining CAT activity.

#### 2.8.3. Guaiacol peroxidase activity (GPX).

To assess GPX enzyme activity, 9.4 µL of extract was mixed with 285 µL phosphate solution (pH 7.0), 4.7 µL H2O2 (3%) and 4.7 µL guaiacol, in microplate reader wells. The plate then was briefly shaken. The alterations in absorbance at 436 nm were monitored for a duration of 1 minute and GPX activity was determined as U g ^− 1^ FW [35].

#### 2.8.4. Ascorbate peroxidase activity (APX).

The measurement of APX activity followed the methodology outlined by Nakano and Asada [36] was conducted using a spectrophotometer at 290 nm. To carry out the assay, 14 µL of extract was mixed with 282 µLof 50 mM phosphate buffer (pH 7.0), 2.8 µL of H2O2 (5%), and 1.4 µL ascorbic acid (50 µM) in tubes. After incubation at 25°C for 1 min, the APX activity was assessed by monitoring the reduction in ascorbate content over a period of 1 minute.

#### 2.8.5. Superoxide dismutase activity (SOD).

For assessment of SOD activity, 10 µL of extract was combined with 290 µL of reaction buffer, consisting of 50 mM phosphate buffer (pH 7), 0.1 M EDTA, 13 mM L-methionine, 4 µM riboflavin and 75 µM NBT in a 96-well plate. Following homogenization, the plates were exposed to light for 15 min, and then the absorbance was assessed at 560 nm. SOD activity was quantified by its ability to reduce NBT photoreduction by 50%, with one unit of SOD activity defined as such [37].

### 2.9. Statistical analysis

Statistical analyses were performed using SAS software (version 9.4). The experiment was arranged as a factorial treatment structure within a completely randomized design with three replicates. Differences among treatment means were evaluated using Fisher’s least significant difference (LSD) test at significance levels of 0.05 and 0.01. In addition, the standard error of the mean was calculated to indicate the precision of the estimates.

### 2.10. Inclusivity in global research

Additional information regarding the ethical, cultural, and scientific considerations specific to inclusivity in global research is included in the Supporting Information (S2 Checklist).

## 3. Results

### 3.1. Hairy root induction and selection of the fastest-growing line

Transgenic hairy roots of Lactuca serriola were successfully induced by inoculating leaf explants from two-week-old seedlings with *Agrobacterium rhizogenes* strain ATCC 15834 (Fig 1). Hairy roots emerged within three weeks at the inoculation site. Among the 12 resulting lines, lines 7 and 12 displayed the fastest and most stable growth. Although line 12 initially showed the highest growth rate, it was lost due to contamination during maintenance culture. Consequently, line 7, which exhibited the second-highest growth rate and a stable 6.76-fold increase in biomass, was selected for all subsequent experiments.

**Fig 1:**
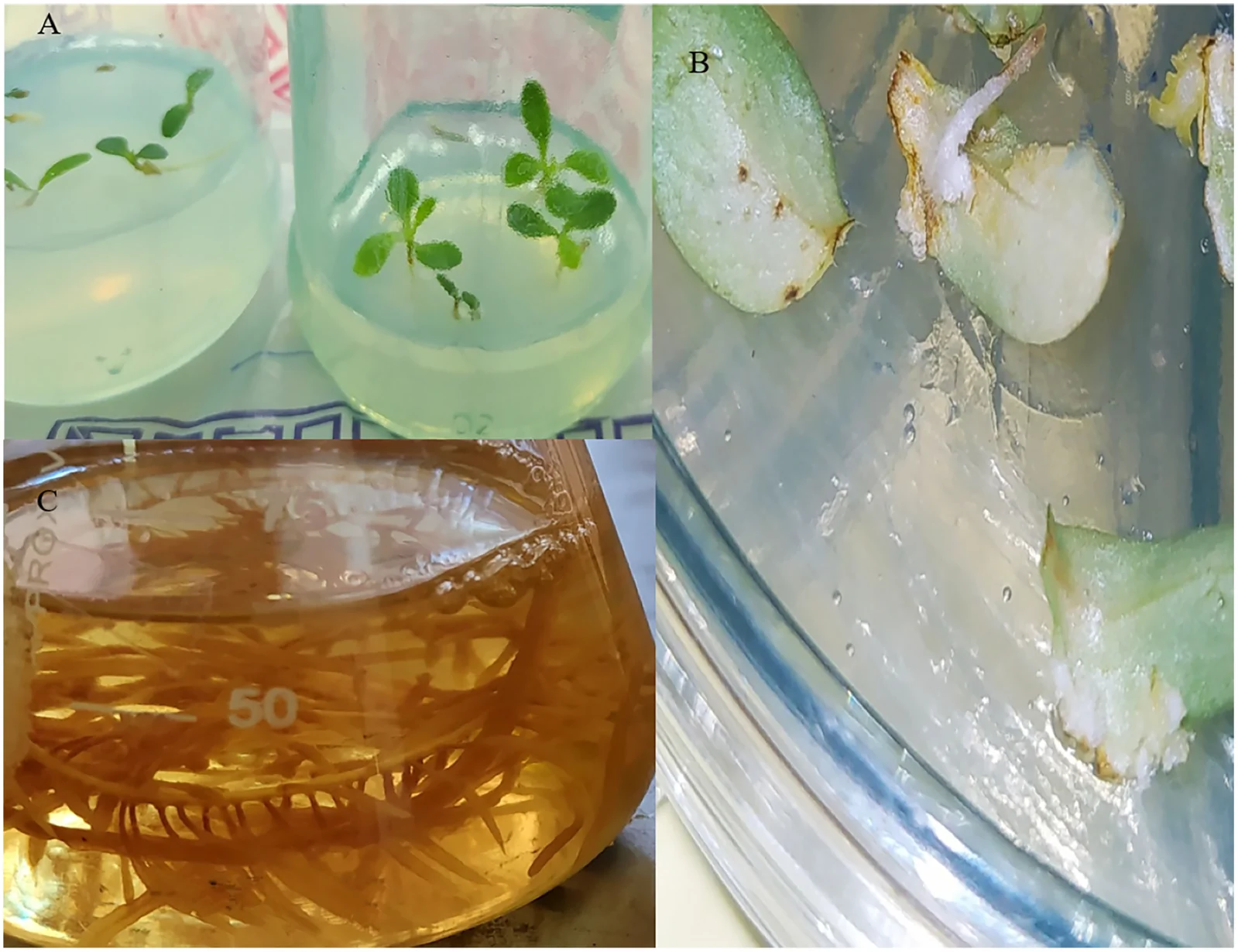
*A. rhizogenes*-mediated transformation of *L.serriola* (A) two weeks old seedlings (B) Hairy root initiation at the wounded sites of leaf explants (C) Hairy roots cultured in liquid ½ MS medium.

### 3.2. PCR analysis for confirmation of *rol C* gene integration

The transgenic nature of the induced hairy roots was confirmed through PCR amplification targeting the *rolC* gene (Fig 2).

**Fig 2:**
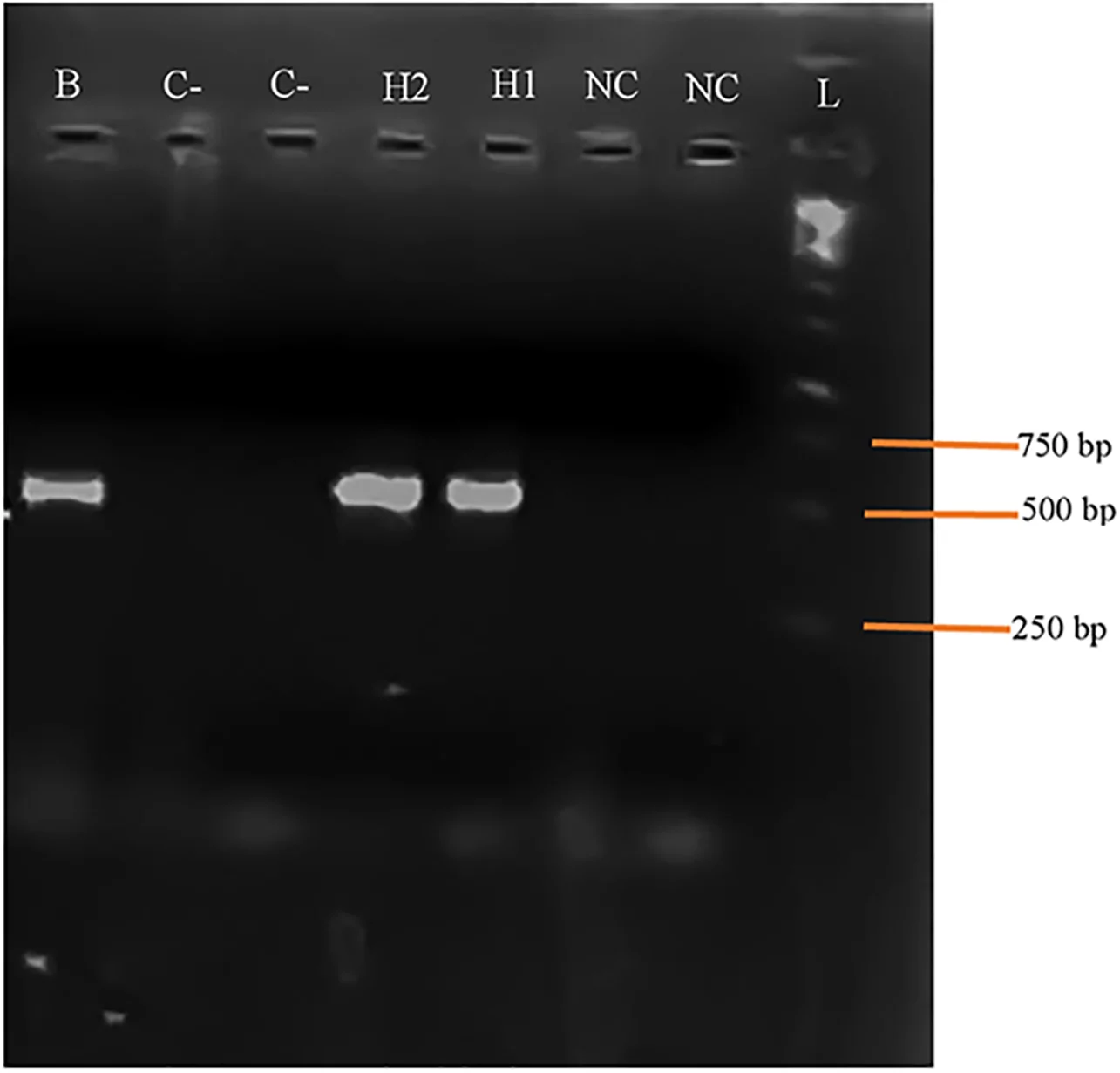
Agarose gel electrophoresis (1%) of PCR products for verification of the presence of the *rolC* gene in hairy roots of *Lactuca serriola.* L: DNA ladder (1 kb); NC: negative control (PCR without template DNA); H1 transgenic line 7; H2: transgenic line 12; C–: non-transformed (normal) root; B: positive control (*A. rhizogenes* ATCC15834); Amplification of the ~ 626 bp *rolC* fragment confirms successful transformation.

PCR amplification yielded the expected ~626 bp fragment corresponding to the *rolC* gene in both transgenic lines tested (Line 7 and Line 12), confirming successful transformation (Fig 2). Line 7 showed consistent amplification, demonstrating the stability of the transgene. The same fragment was amplified from the positive control (*A. rhizogenes* ATCC 15834), while no amplification was observed in the non-transformed root or negative control samples.

### 3.3. Influence of methyl jasmonate on hairy root growth rate

The effects of MeJA concentration and exposure duration on the growth of *Lactuca serriola* hairy roots were evaluated. The interaction between MeJA concentration and exposure time had a statistically significant effect (p < 0.01) on fresh weight. Mean comparison results indicated that the highest fresh weight (3.09 g) was obtained in the control treatment, while the lowest fresh weight (1.18 g) was recorded at 200 µM MeJA after 48 hours (Fig 3). These results demonstrate a decreasing trend in fresh weight with increasing MeJA concentrations. Dry weight was significantly affected by MeJA concentration (p < 0.01), showing a progressive decline with increasing elicitor levels. The highest biomass was observed in control cultures with 0 µM of elicitor (0.28 g), whereas the lowest dry weight (0.11 g) was observed at 200 µM MeJA. In contrast, exposure time and the interaction between time and concentration did not significantly influence dry weight (Fig 4).

**Fig 3:**
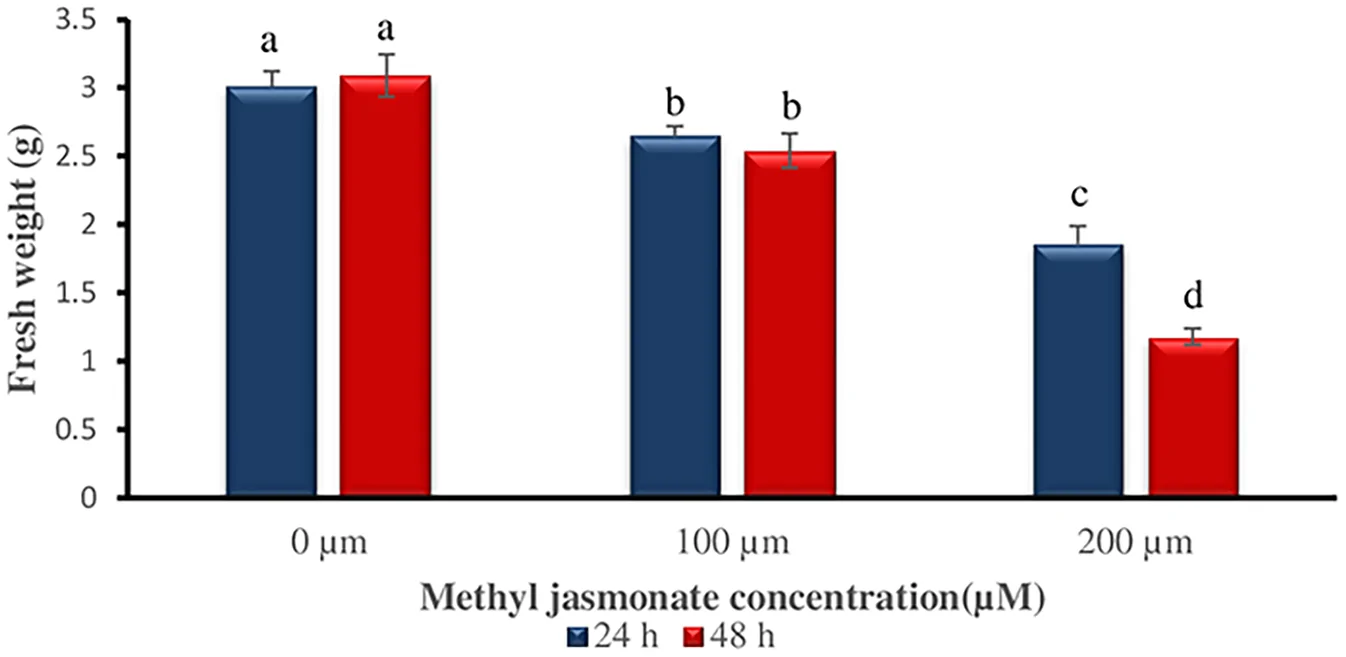
Effect of different concentrations of methyl jasmonate at different exposure times on fresh weight (g) of *Lactuca serriola* hairy roots. Error bars are shown as ±SE (n = 3). Means followed by the same letter are not significantly different according to the Duncan’s multiple range test at p < 0.01.

**Fig 4:**
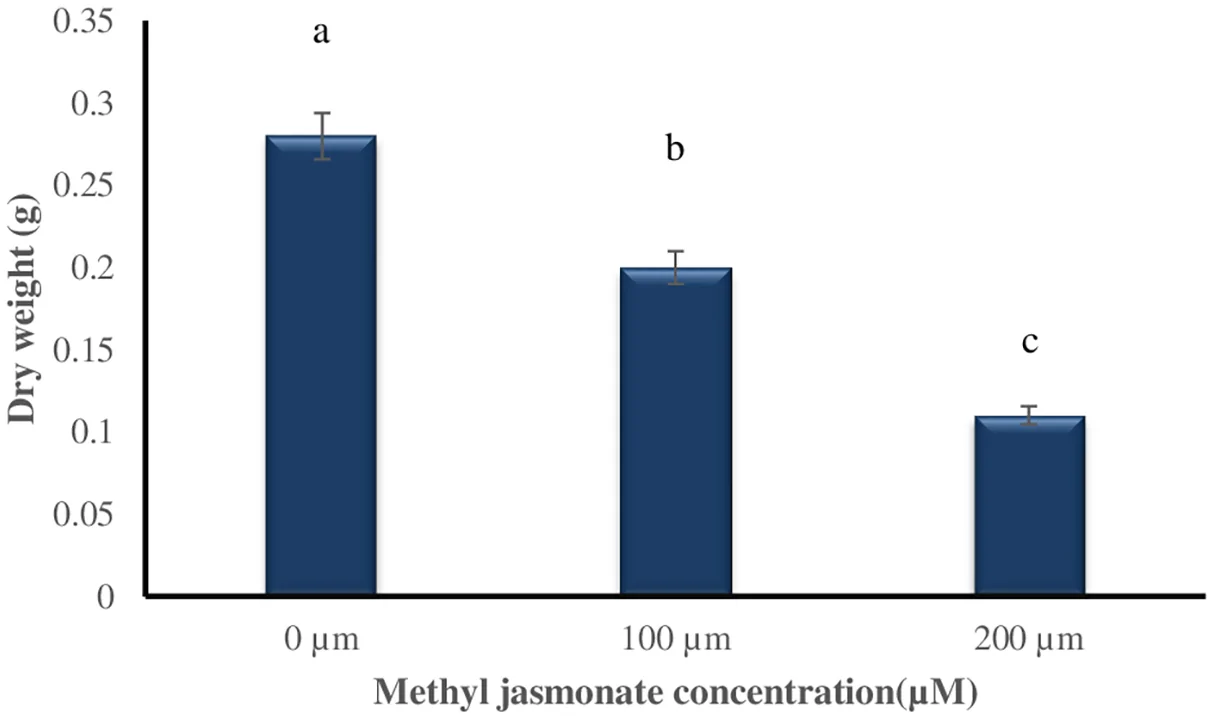
Effect of methyl jasmonate concentration on dry weight of *Lactuca serriola* hairy roots. Error bars are shown as ±SE (n = 3). Means followed by the same letter are not significantly different according to the Duncan’s multiple range test at p < 0.01.

### 3.4. Effect of methyl jasmonate on total phenolic and flavonoid content

MeJA concentration and exposure time significantly affected the TPC of *Lactuca serriola* hairy roots, with a significant interaction at the 1% level. The highest TPC (11.8 mg GAE/g FW) was observed at 200 µM MeJA after 24 hours, while the lowest (6.27 mg GAE/g FW) occurred in the 24-hour control (Fig 5). Overall, MeJA treatments increased TPC across both time points, with the 200 µM treatment showing a 1.9-fold increase over the control.

**Fig 5:**
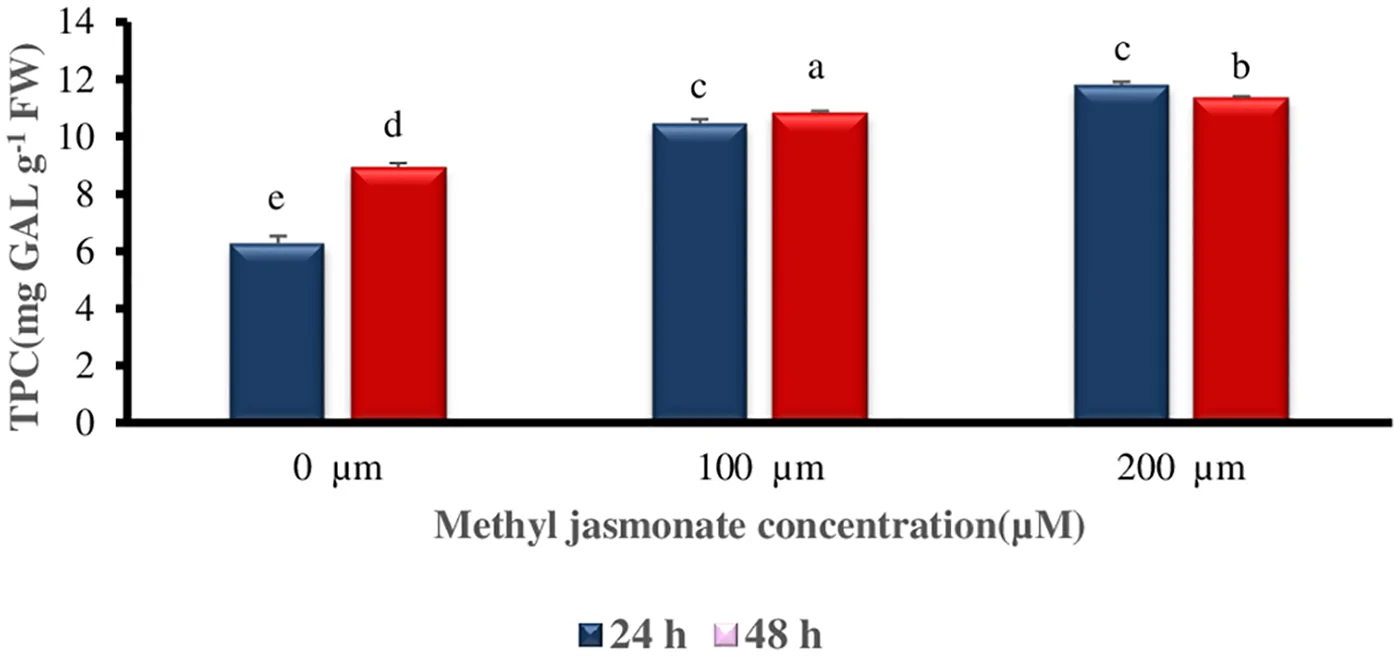
Effect of different concentrations of methyl jasmonate at different exposure times on total phenol content (TPC) (mg Gallic acid g ^– 1^ fresh weight) *Lactuca Serriola* hairy roots. Error bars are shown as ±SE (n = 3). Means followed by the same letter are not significantly different according to the Duncan’s multiple range test at p < 0.01.

For TFC, concentration and treatment duration each had significant effects (p < 0.01), although their interaction was not significant. The highest TFC (6.06 mg QE/g FW) was recorded at 200 µM MeJA, and the lowest (3.37 mg QE/g FW) in the control (Fig 6A). Flavonoid levels were higher after 48 hours (5.31 mg QE/g FW) compared to 24 hours (4.17 mg QE/g FW) (Fig 6B).

**Fig 6:**
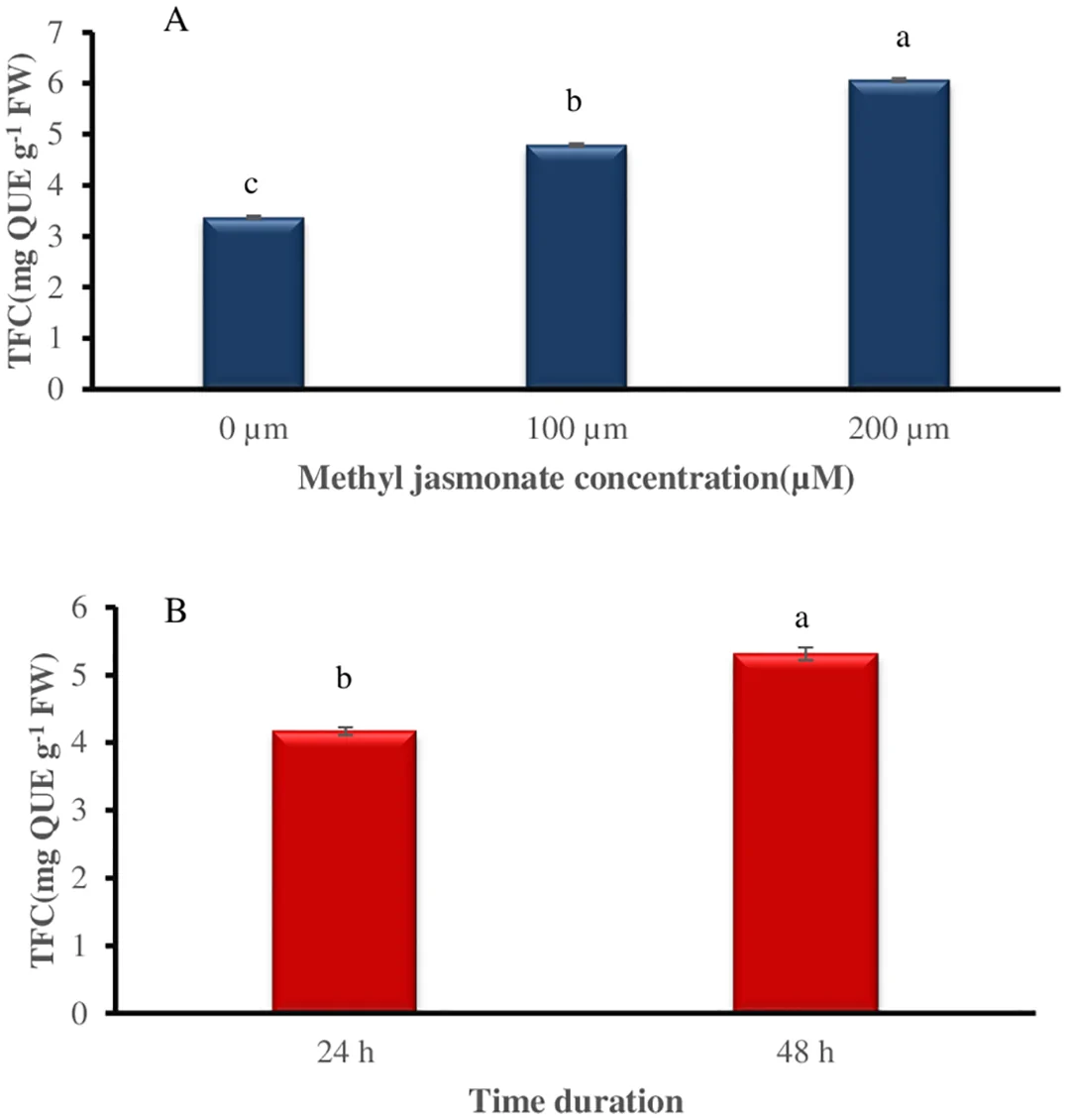
Effect of different concentrations (A) and exposure times (B) of methyl jasmonate on total flavonoid content (TFC) (mg Quercetin g ^– 1^ fresh weight) *Lactuca Serriola* hairy roots. Error bars are shown as ±SE (n = 3). Means followed by the same letter are not significantly different according to the Duncan’s multiple range test at p < 0.01.

### 3.5. Methyl jasmonate effect on antioxidant enzyme activity

MeJA concentration and elicitation time significantly influenced the activity of antioxidant enzymes (SOD, CAT, GPX, APX) in *Lactuca serriola* hairy roots, though their effects were enzyme-specific. A significant interaction between concentration and time was observed for SOD activity (p < 0.01), indicating that the effect of concentration depended on the duration of exposure. The maximum SOD activity (10.05 U/g FW) was detected at 200 µM MeJA after 48 hours, which was 2.3 times higher than the control. Overall, SOD activity increased with increasing MeJA concentration at both 24 and 48 h, peaking at the highest concentration and longer exposure (Fig 7A).

**Fig 7:**
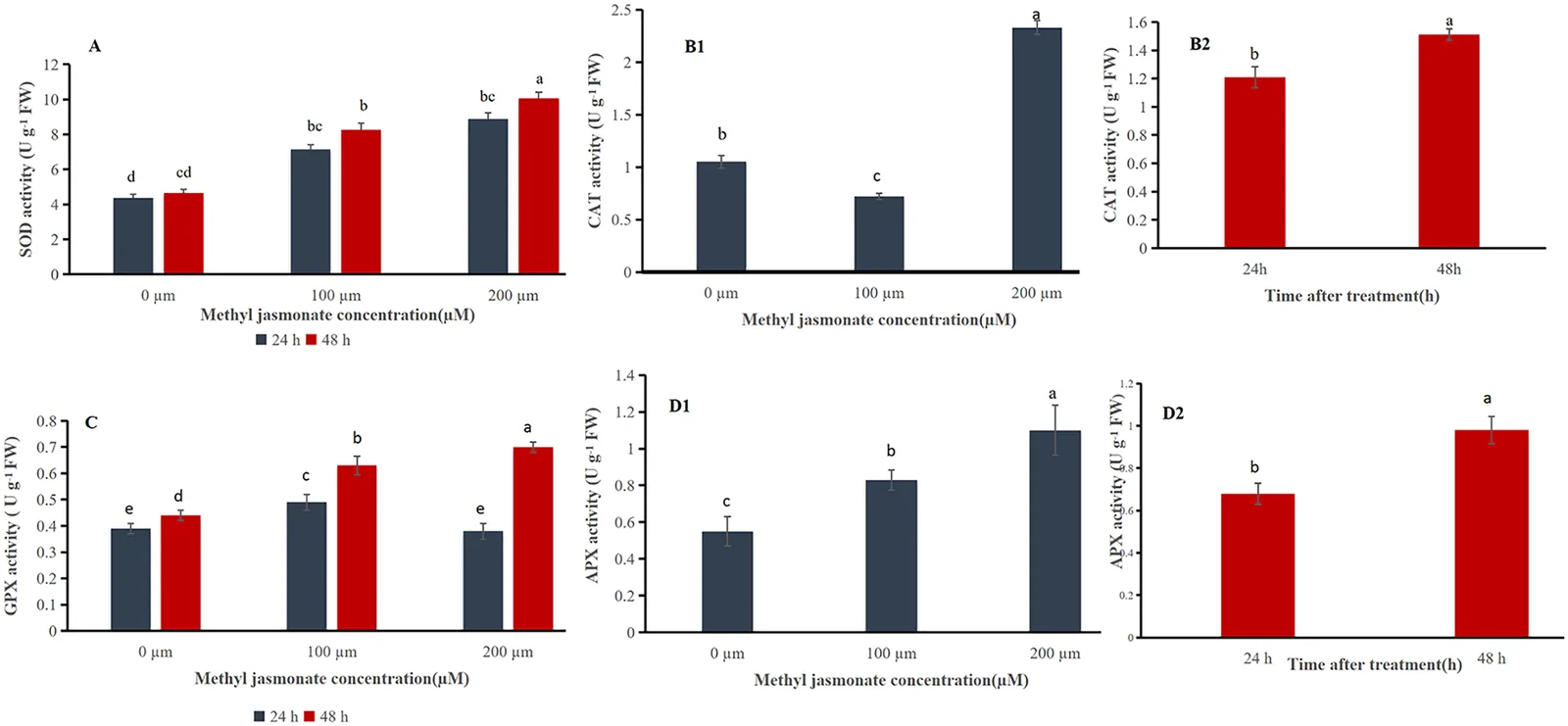
Effect of different concentrations of methyl jasmonate at different exposure times on activity of antioxidant enzymes in *Lactuca Serriola* hairy roots. Error bars are shown as ±SE (n = 3). Means followed by the same letter are not significantly different according to the Duncan’s multiple range test at p < 0.01.

CAT activity was significantly influenced by both MeJA concentration and time individually (p < 0.01), though no significant interaction was observed. The highest CAT activity (2.33 U/g FW) occurred at 200 µM MeJA, while the lowest (0.72 U/g FW) was recorded at 100 µM (Fig. 7B1). Regarding time, CAT activity peaked at 48 h (1.51 U/g FW), with lower activity observed at 24 h (1.21 U/g FW) (Fig.7B2).

GPX activity showed a significant interaction between MeJA concentration and elicitation time (p < 0.01). The maximum GPX activity (0.70 U/g FW) was observed at 200 µM MeJA after 48 h, whereas the lowest (0.38 U/g FW) was found at the same concentration after 24 h. GPX activity generally increased with higher MeJA concentrations (Fig 7C).

APX activity was significantly influenced by both MeJA concentration and exposure time independently (p < 0.01), but not by their interaction. The highest APX activity (1.10 U/g FW) was recorded at 200 µM MeJA, while the lowest (0.55 U/g FW) was found in the untreated control(Fig.7D1). In terms of time, the maximum activity was observed 48 h after elicitation (0.97 U/g FW) (Fig. 7D2).

### 3.6. Effect of methyl jasmonate on individual phenolic compound accumulation

Targeted HPLC analysis revealed that MeJA elicitation induced a strong, dose and time dependent accumulation of phenolic acids and flavonoids in *L. serriola* hairy roots (Table 2). The most pronounced responses were observed for p-coumaric acid, quercetin, and caffeic acid, with maximum levels achieved at 200 µM after 48 h.

**Table 2:** Effect of methyl jasmonate elicitation on phenolic acid and flavonoid contents (μg/g FW) in *Lactuca serriola* hairy root cultures determined by HPLC.

MeJA (µM)	Time(h)	Phenolic acid µg/g					Flavonoid (µg/g)		
		Caf	Chl	Cou	Ros	Cin	Rut	Que	Api
0	24	10.44^e^ ± 0.18	26.32^d^ ± 0.13	19.55^f^ ± 0.32	1.02^d^ ± 0.02	1.35^d^ ± 0.04	1.02^de^ ± 0.05	9.32^d^ ± 0.09	9.31^b^ ± 0.38
	48	11.45^d^ ± 0.2	28.27^c^ ± 0.12	22.38^e^ ± 0.17	1.28^c^ ± 0.01	2.11^c^ ± 0.09	1.3^ab^ ± 0.08	10.7^c^ ± 0.2	9.94^b^ ± 0.18
100	24	8.6^f^ ± 0.14	28.92^c^ ± 0.02	48.32^c^ ± 0.13	1.81^a^ ± 0.04	1.27^d^ ± 0.07	0.9^e^ ± 0.04	11.22^c^ ± 0.08	3.14^d^ ± 0.11
	48	22.6^c^ ± 0.06	27.3^d^ ± 0.1	40.68^d^ ± 0.13	0.46^e^ ± 0.02	4.06^b^ ± 0.06	1.05 cd ± 0.01	7.62^e^ ± 0.14	7.04^c^ ± 0.4
200	24	39.45^b^ ± 0.14	39.28^a^ ± 0.1	61.4^b^ ± 0.24	1.42^b^ ± 0.01	2.25^c^ ± 0.1	1.16^bc^ ± 0.04	22.76^b^ ± 0.11	10.13^b^ ± 0.18
	48	46.7^a^ ± 0.18	37.2^b^ ± 0.35	78.64^a^ ± 0.19	0.38^f^ ± 0.02	8.36^a^ ± 0.14	1.41^a^ ± 0.01	49.56^a^ ± 0.27	20.87^a^ ± 0.27

MeJA: methyl jasmonate; Caf: caffeic acid; Chl: chlorogenic acid; Cou: coumaric acid; Ros: rosmarinic acid; Cin: cinnamic acid; Rut: rutin; Que: quercetin; Api: apigenin. Values represent the means of three replicates. Different letters within a column indicate significant differences according to Duncan's multiple range test at p < 0.01.

P-Coumaric acid accumulation was particularly remarkable, reaching 78.64 µg/g FW (3.5-fold over control) at 200 µM after 48 h and 61.4 µg/g FW at 24 h. A significant interaction between MeJA concentration and exposure time (p < 0.01) indicated that higher doses amplified the effect over time. At 100 µM, moderate increases were also observed.

Quercetin followed a similar pattern, peaking at 49.56 µg/g FW (4.6-fold over control) at 200 µM after 48 h, with a significant concentration × time interaction (p < 0.01). Caffeic acid reached 46.7 µg/g FW at the same treatment, representing a 4.1-fold induction, and was significantly affected by both factors (p < 0.01).

Chlorogenic acid content peaked earlier, at 39.28 µg/g FW (1.5-fold) after 24 h at 200 µM, declining slightly to 37.2 µg/g FW at 48 h. Apigenin showed a significant interaction (p < 0.01), with maximum accumulation (20.87 µg/g FW, 2.1-fold) at 200 µM after 48 h.

Cinnamic acid exhibited consistent, concentration-dependent increases, reaching 8.36 µg/g FW (4.0-fold) at 200 µM after 48 h. Rutin accumulation was modest (1.41 µg/g fW maximum, 1.1-fold), with significant main effects of time and concentration but no interaction.

In contrast, rosmarinic acid displayed a transient response, peaking at 1.81 µg/g FW with 100 µM after 24 h before declining sharply (p < 0.01), while 200 µM induced only slight early increases followed by decreases.

Overall, among the conditions tested, treatment with 200 µM MeJA for 48 h resulted in the highest accumulation of most phenolic compounds, particularly early phenylpropanoid derivatives and flavonols.

## 4. Discussion

The genus *Lactuca* is a valuable system for investigating the regulation of phenolic metabolism and stress-responsive secondary metabolites, although these responses remain relatively underexplored [38,39]. In this context, hairy root cultures provide a stable and controllable platform for investigating plant secondary metabolism under defined experimental conditions [40]. To the best of our knowledge, this study is the first to comprehensively characterize MeJA-induced phenolic accumulation and antioxidant responses in *L. serriola* hairy roots. MeJA treatment markedly influenced both growth and secondary metabolism, enhancing antioxidant enzyme activities and stimulating the accumulation of several phenolic acids and flavonoids while simultaneously reducing biomass. These findings indicate a jasmonate-driven metabolic shift from primary growth toward defense-related pathways and highlight the suitability of *L. serriola* hairy roots as an effective *in vitro* model for investigating elicitor-induced regulation of phenolic metabolism and antioxidant defense.

Hairy roots were successfully induced within 3–4 weeks following infection of leaf explants with *Agrobacterium rhizogenes*, although considerable variation in growth performance was observed among independently transformed lines. Such variability is commonly observed among independently transformed hairy root lines and is generally attributed to differences in T-DNA integration sites, copy number, and the expression of *rol* genes encoded on the Ri plasmid, which collectively influence endogenous phytohormone balance, root architecture, and secondary metabolism [41,42]. Although Line 12 initially exhibited the highest growth rate, it was lost because of contamination during maintenance culture. Consequently, Line 7, which showed vigorous growth and a stable 6.76-fold increase in biomass, was selected for subsequent elicitation experiments. PCR analysis confirmed the stable integration of the *rolC* gene, verifying the successful genetic transformation of *Lactuca serriola* hairy roots [43,44].

MeJA elicitation caused dose and time-dependent reductions in fresh and dry biomass, particularly under 200 µM treatment and prolonged exposure. Such growth inhibition is consistent with the well-established growth–defense trade-off, whereby jasmonate signaling reallocates cellular resources from primary growth to defense-related metabolism [45,46]. The reduction in biomass may also result from jasmonate-mediated modulation of auxin-related growth pathways, which can restrict cell expansion during stress adaptation [47–49]. In parallel with the reduction in biomass, MeJA-treated hairy roots exhibited enhanced antioxidant enzyme activities and increased accumulation of phenolic metabolites, suggesting that activation of defense-related metabolism occurred at the expense of growth. Similar responses have been reported in MeJA-elicited hairy root cultures of *Ficus carica* and *Echinacea purpurea*, where comparable trade-offs between biomass accumulation and secondary metabolite production were observed, highlighting the importance of optimizing elicitor concentration and exposure duration [47,50].

MeJA elicitation triggered a coordinated activation of the antioxidant defense system in *L. serriola* hairy roots, as demonstrated by significant increases in the activities of SOD, CAT, GPX and APX. The highest enzyme activities were observed after 48 h of treatment with 200 μM MeJA, coinciding with the greatest accumulation of total phenolics, total flavonoids, and several individual phenolic compounds. These enzymes constitute an integrated ROS-scavenging network that maintains cellular redox homeostasis by converting ROS into less reactive molecules, thereby limiting oxidative damage while allowing ROS to function as essential signaling molecules during stress adaptation [48]. The simultaneous enhancement of antioxidant enzyme activities and phenolic accumulation suggests that MeJA-induced ROS signaling promotes metabolic adjustment toward defense-associated pathways, particularly phenylpropanoid biosynthesis. Thus, ROS generated during MeJA perception appear to act not only as potential oxidative stressors but also as secondary messengers that coordinate antioxidant protection and specialized metabolism. This integrated response highlights the role of jasmonate signaling in linking redox regulation with the activation of plant defense mechanisms. Similar coordination between jasmonate-mediated signaling, antioxidant enzyme activation, and phenolic metabolism has been reported in hairy root cultures and intact plant systems, supporting the conserved role of MeJA in regulating stress-responsive metabolic pathways [3,6,51].

In addition to activating enzymatic antioxidant defenses, MeJA elicitation markedly enhanced the accumulation of phenolic compounds in *L. serriola* hairy roots, as reflected by increased total phenolic content (TPC) and total flavonoid content (TFC). The highest TPC and TFC levels were observed following treatment with 200 µM MeJA, indicating a strong stimulation of phenylpropanoid metabolism. Jasmonates are well known regulators of secondary metabolism, inducing the expression and activity of key enzymes involved in phenylpropanoid biosynthesis, including phenylalanine ammonia-lyase (PAL), which catalyzes the entry step of this pathway [45,52,53]. The accumulation of these non-enzymatic antioxidants likely contributes to enhanced stress tolerance by providing additional protection against oxidative challenges generated during elicitor-induced defense activation. However, the magnitude of phenolic accumulation depends strongly on elicitor concentration, exposure duration, and species-specific metabolic responses, as excessive MeJA levels may impose metabolic costs associated with stress responses and growth inhibition [54–56]. Similar increases in phenolic and flavonoid compounds following MeJA application have been reported in *Ficus carica* cv. Siah, *Salvia viridis*, and finger millet sprouts, demonstrating the conserved role of jasmonate signaling in stimulating phenylpropanoid-derived defense metabolites across diverse plant systems [41,47,57,58].

The coordinated responses observed in the present study indicate that MeJA does not simply stimulate the accumulation of individual metabolites but rather induces a broader metabolic reprogramming associated with plant defense. The simultaneous reduction in biomass, activation of enzymatic antioxidant systems, and enhanced accumulation of total phenolics and flavonoids suggest that jasmonate signaling redirects metabolic resources toward the secondary metabolism while maintaining cellular redox homeostasis. Such coordinated regulation enables plants to respond efficiently to stress by integrating ROS signaling with the biosynthesis of protective secondary metabolites. This coordinated response provides a physiological framework for interpreting the compound-specific changes revealed by HPLC analysis.

Compound-specific HPLC profiling demonstrated that MeJA did not uniformly enhance all phenylpropanoid metabolites but instead selectively redirected metabolic flux toward specific branches of the pathway. Following treatment with 200 µM MeJA for 48 h, p-coumaric acid, quercetin, and caffeic acid exhibited the greatest increases, whereas rutin and rosmarinic acid accumulated only at low levels. The substantial accumulation of p-coumaric acid and caffeic acid suggests enhanced flux through the upstream phenylpropanoid pathway, while the pronounced increase in quercetin indicates preferential activation of flavonoid biosynthesis under MeJA elicitation. In contrast, the limited accumulation of rosmarinic acid despite elevated caffeic acid concentrations may reflect restricted activity of downstream biosynthetic enzymes, limited precursor channeling, or species-specific regulation of this pathway branch in *L. serriola* hairy roots [12]. Such differential metabolite accumulation illustrates that MeJA regulates not only the overall production of phenolic compounds but also their partitioning among individual biosynthetic branches. Similar metabolite-specific responses have been reported in other hairy root cultures. For example, MeJA increased rosmarinic acid and prolithospermic acid accumulation in *Salvia viridis* [41], whereas hairy roots of *Ficus carica* cv. Siah exhibited marked increases in several phenolic acids in a concentration- and time-dependent manner [47].

Collectively, these observations indicate that activation of the specialized metabolism is a conserved response to MeJA elicitation, although the magnitude and composition of accumulated metabolites are highly species dependent. The contrasting accumulation patterns observed among individual phenolic compounds suggest that MeJA selectively regulates specific branches of the phenylpropanoid pathway rather than uniformly enhancing overall phenolic metabolism. This selective response likely reflects differential regulation of downstream biosynthetic enzymes, which ultimately shapes the metabolite profile of *L. serriola* hairy roots [48].

While the present study demonstrates the effectiveness of MeJA elicitation in modifying phenolic metabolism in *L. serriola* hairy roots, several limitations should be considered. The reduction in biomass observed at higher MeJA concentrations indicates that elicitation conditions should be optimized to balance biomass accumulation with metabolite production. In addition, although hairy root cultures provide a valuable model for investigating specialized metabolism, their responses may not fully represent the complexity of whole-plant physiological regulation. The relatively limited accumulation of rosmarinic acid further suggests that additional elicitation strategies, precursor feeding, or optimization of culture conditions could be explored to enhance metabolite accumulation through this branch of the secondary metabolism. Finally, future studies integrating metabolite profiling with transcriptomic, proteomic, or enzyme activity analyses would provide a more comprehensive understanding of the molecular mechanisms governing MeJA-induced metabolic regulation in *L. serriola*.

## 5. Conclusion

To our knowledge, this study provides the first comprehensive evaluation of methyl jasmonate-induced changes in growth, antioxidant responses, and phenolic metabolism in *Lactuca serriola* hairy root cultures. MeJA elicitation significantly altered growth, enhanced antioxidant enzyme activities, and promoted the accumulation of phenolic acids and flavonoids, demonstrating coordinated regulation of antioxidant defense and phenylpropanoid metabolism during jasmonate signaling. Although higher MeJA concentrations reduced biomass, they also promoted the characteristic growth–defense trade-off by redirecting cellular resources toward defense-associated secondary metabolism. These findings improve our understanding of jasmonate-mediated regulation of phenylpropanoid metabolism in transformed roots and establish *L. serriola* hairy root cultures as a valuable experimental system for investigating stress-responsive specialized metabolism under controlled in vitro conditions. The knowledge generated in this study provides a foundation for future investigations aimed at optimizing elicitation strategies and further exploring the regulation of specialized metabolite biosynthesis in plant tissue cultures.

## Supporting information

S1 Fig Original uncropped and unadjusted gel image. Original gel image corresponding to Fig 2, provided to show the complete gel before cropping and image adjustment.
(PDF)

S2 Cheklist Inclusivity in global research. (DOCX)
