# Supplementary material for: Establishment of hairy root cultures of Lactuca serriola and enhancement of phenolic metabolites through methyl jasmonate elicitation
Source: PLoS One. 2026 Nov 4;21(11):e0360818. doi: 10.1371/journal.pone.0360818 (PMC13734474; doi:10.1371/journal.pone.0360818)
Supplement: Supplementary file 1 [file pone.0360818.s001.pdf]

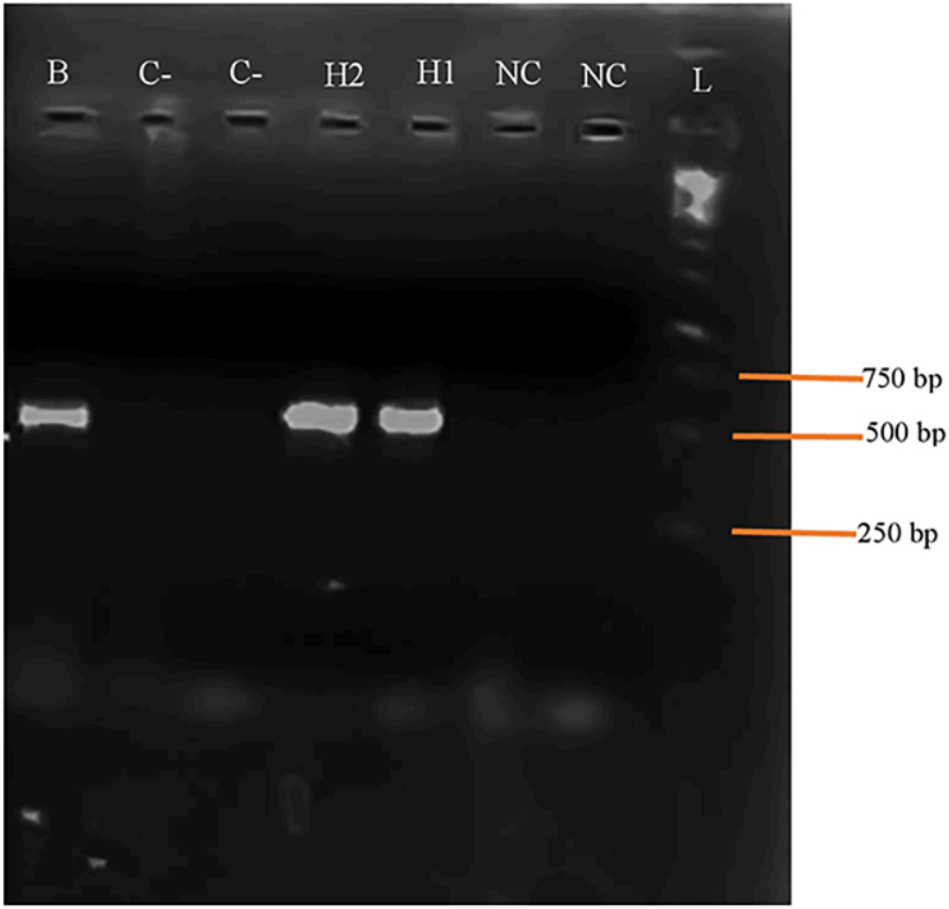
B
C-
C-
H2
H1
NC
NC
L
750 bp
500 bp
250 bp

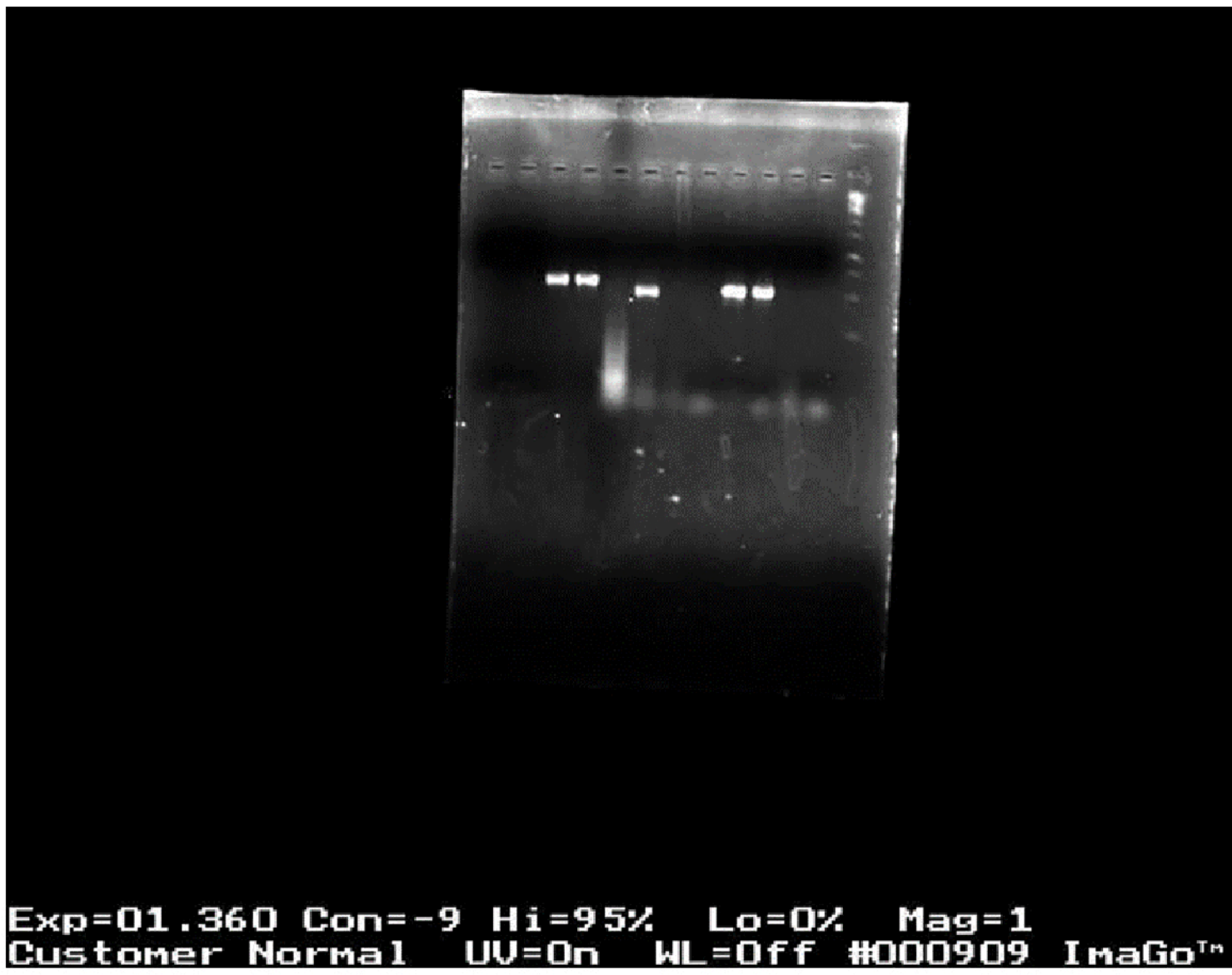

Figure 2 – Original uncropped and unadjusted gel image
Corresponding manuscript figure: Fig. 2
Image acquisition: Agarose gel image captured using gel doc instrument.

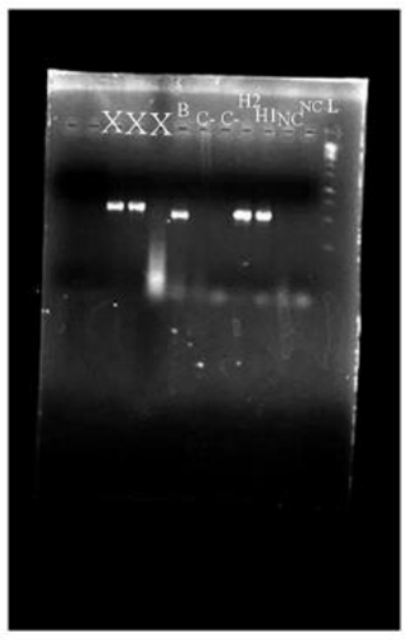

L: 1 kb DNA ladder.
NC: negative control (PCR without template DNA)

H1: hairy root line

H2: hairy root line

C-: normal root

X: lane not included in the final Fig. 2
